# High productivity of oxylipin KODA using *E. coli* transformed with lipoxygenase and allene oxide synthase genes of *Lemna paucicostata*

**DOI:** 10.5511/plantbiotechnology.24.0721a

**Published:** 2024-12-25

**Authors:** Kazuteru Takagi, Mineyuki Yokoyama, Toshio Beppu, Haruna Uemori, Hirokazu Ohno, Toshiyuki Murakami, Ohji Ifuku, Yuichi Tada, Shigeo Yoshida

**Affiliations:** 1 Shiseido Research Center, Hayabuchi 2-2-1, Tsuzuki, Yokohama, Kanagawa 224-8558, Japan; 2 School of Bioscience and Biotechnology, Tokyo University of Technology, 1404-1, Hachioji, Tokyo 192-0982, Japan; 3 Faculty of Education & Human Science, Teikyo University of Science, 2525 Yatsusawa, Uenohara, Yamanashi 409-0193, Japan; 4 Research and Development Division, Maruzen Pharmaceuticals Co., Ltd., 1089-8, Sagata Shin-ichi, Fukuyama, Hiroshima 729-3102, Japan; 5 RIKEN Plant Science Center, 1-7-22, Suehiro-cho, Turumi Ward, Yokohama, Kanagawa 230-0045, Japan

**Keywords:** allene oxide synthase, *E. coli*, KODA, *Lemna paucicostata*, lipoxygenase

## Abstract

KODA, a type of oxylipin, has stimulatory effects on plant growth under limiting conditions of stress, such as promoting flowering, rooting, and resistance to pathogens, for use in agriculture. KODA is released from *Lemna paucicostata* under drought, heat, and osmotic pressure, and is produced from α-linolenic acid by a two-step enzymatic reaction with 9-lipoxygenase and allene oxide synthase. In this paper, we report the outstanding KODA productivity of *L. paucicostata*, SH strain screened from 56 *Lemna* species. We constructed co-expression vectors for 9-lipoxygenase gene (*LpLOX*) and allene oxide synthase gene (*LpAOS*) isolated from the SH strain to be transformed into *E. coli*. The productivity (per fresh weight) using *E. coli* is 25.3 mg KODA g^−1^ as compared to 0.366 mg g^−1^ from *L. paucicostata*, SH strain, which requires a longer culture time, light irradiation and larger space for culture.

KODA, an oxylipin, is generated from linolenic acid by 9-lipoxygenase, while jasmonic acid in the same group is synthesized by 13-lipoxygenase (Yokoyama 2005). Oxylipins could be produced when plants are exposed to abiotic stress, such as drought, cold and ozone (Ali and Baek 2020) because phospholipase is activated by abiotic stresses to detach linolenic acid (Iqbal et al. 2020). KODA is released from *Lemna paucicostata* subjected to stresses such as drought, heat and osmotic pressure (Takimoto et al. 1994; Yokoyama et al. 2000). Both KODA and jasmonic acid, are stress-related components, but have different activities. KODA reacting with norepinephrine showed strong flower-inducing activity in *Lemna paucicosta*, 151 strain (Yamaguchi et al. 2001; Yokoyama et al. 2008), but jasmonic acid did not (Yokoyama et al. 2000). KODA shows various promotive effects on growth in plants under marginal conditions of stress as described below, while jasmonic acid generally suppresses growth and promotes tolerance in plants under stresses (Wasternack and Hause 2013).

KODA was initially shown to promote flowering in various plants such as *Lemna paucicostata* (Yokoyama et al. 2000), *Malus domestica* Borkh. (Kittikorn et al. 2010, 2011), *Citrus unshiu* (Nakajima et al. 2011, 2016), and *Pharbitis nil* (Ono et al. 2013). KODA also promotes rooting (Kawakami et al. 2015), re-shooting after dormancy (Nakajima et al. 2016; Sakamoto et al. 2010, 2012). It also increases wheat yield (Haque et al. 2016) and enhances the resistance of certain herbaceous plants to pathogens (Endo et al. 2013; Wang et al. 2016). These findings suggest the potential usefulness of KODA in agriculture.

KODA was originally found as the component released from *L. paucicostata* when subjected to stress, such as drought, heat or osmotic pressure (Takimoto et al. 1994; Yokoyama et al. 2000). We suspected that there is a *Lemna* species producing much more KODA than the 441 strain we used in the experiment. We examined the productivity of KODA over 17 species of *L. paucicostata*, 15 species of *L. aequioctialis* and 24 species of *L. minor*, which were maintained at Teikyo University of Science, although the classification of the species belonging to *Lemna paucicostata* is still disputed (Beppu and Takimoto 1981; Beppu et al. 1985). Any *Lemna* species released KODA after draught stress, however among them, we found one species, we named SH strain, which was a prominent producer of KODA (Figure 1). We devised a strategy to produce KODA using *E. coli*, which would be more efficient than culturing *Lemna* species. KODA is synthesized from linolenic acid by two enzymes, 9-specific lipoxygenase and allene oxide synthase (Yokoyama 2005). We previously identified the genes of lipoxygenase (*LpLOX*, HV534700 in the NCBI database; Yokoyama et al. 2011b) and allene oxide synthase (*LpAOS*, HV534712 in the NCBI database; Yokoyama et al. 2011a) from the novel strain of *L. paucicostata*, cv. SH. *LpLOX* has slightly higher enzymatic activity for KODA production than *Oryza sativa* 9-lipoxygenase (*r9-LOX1*), judging from *K*_m_, *V*_max_ and *k*_cat_ values (Mizuno et al. 2003; Yokoyama et al. 2011b). Furthermore, *LpAOS* has higher enzymatic activity than *Arabidopsis* *AOS* (*AtAOS*) (Kubigsteltig et al. 1999), which we used for KODA production before using *LpAOS*: the *K*_m_ value is 5 times lower, and *V*_max_ and *k*_cat_ are about 3 times higher (Yokoyama et al. 2011a). *LpLOX* and *LpAOS* may produce large amounts of KODA. *E. coli* transformed with *LpLOX* and *LpAOS* would be efficient producers of KODA.

**Figure 1:**
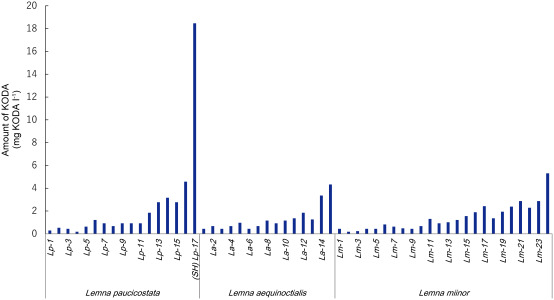
Figure 1. Comparison of KODA productivity on various *Lemna* species. 17 species of *Lemna paucicostata*, 15 species of *Lemna aequinoctialis* and 24 species of *Lemna minor* were analyzed on KODA productivity, which had been stocked in Teikyo University of Science. Each plant of 50 mg in fresh weight was spread on dry filter paper and kept for 30 min at room temperature, followed by immersion in 1 ml distilled water for 2 h. The water in which the plants were immersed was analyzed with HPLC on such condition as, column; Capcell pak C18 (250×4.5 mm I. D., Shiseido Co. Ltd., Tokyo, Japan) at 30°C, eluent; 50% acetonitrile containing 0.1% trifluoro acetic acid, monitor; at 210 nm, flow rate; 1 ml min^−1^. KODA was detected at ca. 14 min.

To reduce the manufacturing costs, we simplified the manufacturing method for the preparation of *LpLOX* and *LpAOS*. RNA extraction and cDNA synthesis from *L. paucicostata*, cv. SH were performed according to the method of Yokoyama et al. (2011a). To prepare the *LpLOX* and *LpAOS* enzymes, we constructed the co-expression vector of the *LpLOX* and the *LpAOS* using the pETDuet-1 vector (Novagen, Madison, WI). We prepared the expression plasmid for the *LpLOX* and the *LpAOS* cDNA using In-Fusion™ Advantage PCR Cloning Kit w/Cloning Enhancer (Clontech Laboratories, Inc., Palo Alto, CA). The primer carrying the recombination site (bold type), for the *LpLOX* cloning was as follows: *LpLOX*-pETDuet-1-For, 5′-AGG AGA TAT ACC ATG GCC GGT TTT CTC CAA AAG G-3′. The pair of primers were as follows: *LpLOX*- pETDuet-1-For and *LpLOX*-pETDuet-1-Rev, 5′-AAG CAT TAT GCG GCC GCT CAA ATG GAG ATG CTG TTG GGG-3′. The primer carrying the recombination site (bold type), for the *LpAOS* cloning was as follows: *LpAOS*-pETDuet-1-For, 5′-AAG GAG ATA TAC ATA TGT CTG TCT CGC AAT CAG ATG-3′. The pair of primers were as follows: *LpAOS*- pETDuet-1-For and *LpAOS*-pETDuet-1-Rev, 5′-GTG GCA GCA GCC TAG TTA TCG GGT CGT CGC CTT CG-3′. PCR products were generated by using PrimeSTAR® HS (TaKaRa) DNA polymerase and purified 0.8% agarose gel. The pETDuet-1 expression vector was linearized by digestion with NcoI (New England Biolabs, Inc., Beverly, MA) and NotI (New England Biolabs, Inc.). The *LpLOX* PCR product was cloned into linearized pETDuet-1 (Novagen) expression vector by using In-Fusion™ Advantage (Clontech) according to the manufacturer’s instruction. Then, the pETDuet-1 (Novagen) expression vector with *LpLOX* introduced was linearized by digestion with NdeI (New England Biolabs, Inc., Beverly, MA) and AvrII (New England Biolabs, Inc.). The *LpAOS* PCR product was cloned into linearized pETDuet-1 (Novagen) expression vector with *LpLOX* introduced by using In-Fusion™ Advantage (Clontech). The resulting expression plasmid vector (Figure 2) was introduced into the expression host *E. coli* BL21 (DE3) (Novagen).

**Figure 2:**
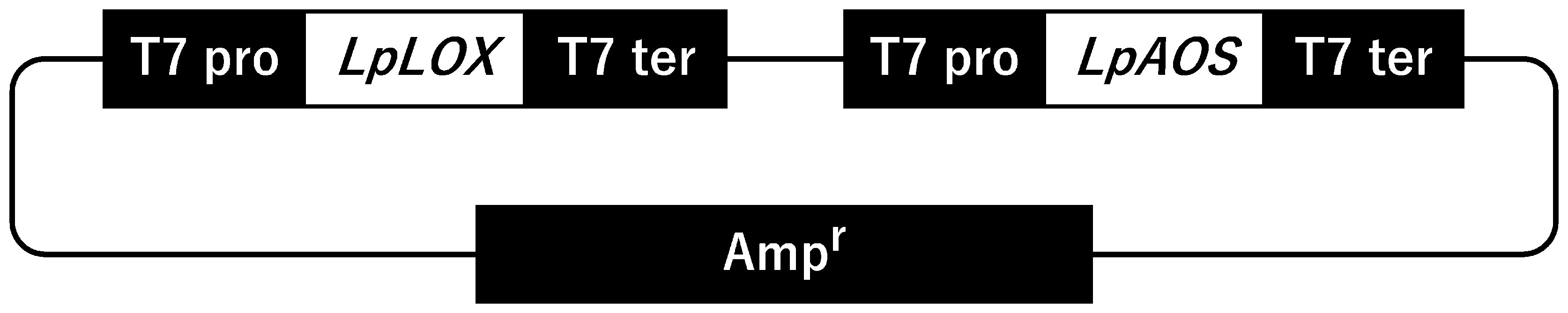
Figure 2. *LpLOX* and *LpAOS* co-expression vectors. Schematic illustration of the plasmid used for transformation of *E. coli*. Gene expression of *LpLOX* and *LpAOS* is regulated by the T7 promoter, respectively. Amp^r^, ampicillin-resistance gene.

The BL21 (DE3) harboring the plasmid was incubated in TB medium containing 100 mg l^−1^ carbenicillin at 37°C as preculture until OD_600_ nm became 1.5. After IPTG (0.1 mM final concentration) was added, the expression procedure was carried out at 15°C, 250 rpm, until OD_600_ nm 20. After the culture was completed, *E. coli* was collected by centrifugation. For protein extraction, 200 µl (5 times the weight of bacteria) of BugBuster™ (Novagen) was added to 40 mg of wet bacterial cell weight to lyse the bacterial cells. Thereafter, the supernatant was collected by centrifugation (20,000×g, 5 min, 4°C) and used as a crude enzyme solution. The composition of the reaction solution was prepared by the following method so that the final concentration was α-linolenic acid 5 mM, Tween 80 0.1%, and phosphate buffer 40 mM (pH 6.5). After mixing 15 µl of α-linolenic acid and 10 µl of Tween 20, a stock solution of the substrate was prepared with milli Q water to a total volume of 1,000 µl (10×LNA solution). After stirring well, 200 µl of 10×LNA solution was taken, the total volume was made up to 1000 µl with milli Q water, and then sonicated (28 Hz, 5 min) while cooling on ice to obtain a 2×LNA solution. Then 200 µl of 200 µM phosphate buffer (pH 6.5) was added to 500 µl of 2×LNA solution, the total volume was made 900 µl with milli Q water. The solution was mixed well by pipetting, and used as the KODA reaction solution. Then 100 µl of the prepared crude enzyme solution was added to the KODA reaction solution, the solution was stirred well by pipetting, and then incubated at 25°C for 30 min. Then 10 µl of the reaction solution was directly analyzed by HPLC. When *E. coli* was added directly to the reaction solution, 200 µl of 200 µM phosphate buffer (pH 6.5) was added to 500 µl of 2×LNA solution, and the total volume was made 1,000 µl with milli Q water, and mixed well by pipetting. The solution was stirred and used as the KODA reaction solution. After being cultured, *E. coli* was collected by centrifugation, 1,000 µl of KODA reaction solution was added to 20 mg of wet bacterial cell weight, the solution was stirred well by pipetting, and incubated at 25°C for 30 min. Then 10 µl of the reaction solution was directly analyzed by HPLC. When preparing the crude enzyme extract and when *E. coli* was added directly to the reaction solution, the added enzyme and *E. coli* were adjusted to be equivalent by calculating the amount of *E. coli* used for the preparation. When the crude enzyme was prepared from *E. coli* that co-expressed *LpLOX* and *LpAOS* and after reaction, the KODA concentration in the reaction solution was 773±25.6 µM (*n*=4). On the other hand, when *E. coli* cells co-expressing *LpLOX* and *LpAOS* were collected and directly added to the reaction solution, the KODA concentration in the reaction solution was 1,630±127 µM (*n*=3). By directly using *E. coli* co-expressing *LpLOX* and *LpAOS*, the KODA productivity was improved two-fold. The productivity (per fresh wight) of this method using *E. coli* is 25.3 mg KODA g^−1^ as compared to 0.366 mg g^−1^ from *L. paucicostata*, SH strain, which requires a longer culture time, light irradiation, and larger space for culture.

Isolated KODA was analyzed with LC-tofMS (Xevo G2 Tof, Waters, Massachusetts, USA) using column: ACQUITY UPLC BEH C_18_ 1.7 µm (2.1×150 mm, Waters), column temperature: 40°C, solvent: MeCN/HCOOH=1,000/1 for analysis and water/HCOOH=1,000/1 for washing, flow rate: 0.3 ml min^−1^, injection volume: 1.0 µl, ionization source: ES negative. LC-MS analysis detected the fragments, *m*/*z* 309 [M-H]^−^, 291 [M-H-H_2_O]^−^, 265 [M-COOH]^−^, 247 [M-COOH-H_2_O]^−^. The fragments of 309 and 291 were identified with the results of LC-MS/MS analyses reported by Suzuki et al. (2003). ^1^H-NMR and ^13^C-NMR profiles were identified with the data reported by Yokoyama et al. (2000).

In summary, we have developed a basic process for KODA production that solves the limitations on culture time and culture space and simplifies the production process to further reduce the cost. KODA production has been reported possible via ectopic expression in plants, using *LpLOX* and *LpAOS*, the same gene as we used here (Ihara et al. 2022). In that case, specific sub-cellular localization, and incubation of crude leaf extracts, which liberates α-linolenic acid via breakdown of endogenous lipids. This report is characterized by directly adding the substrate, α-linolenic acid, and using *E. coli*, which is frequently used in industry.
